# Editorial and Miscellaneous

**Published:** 1864-01

**Authors:** 


### C. S. Medical & Surgical Journal.

RICHMOND, JANUARY, 1864.

*AYRES & WADE.................................PUBLISHERS.*

*Salutatory.*

The Confederate States Medical and Surgical Journal, on making its first appearance before the public, might be expected, under usual circumstances, to congratulate itself and its readers on the new bonds of union and sympathy about to be established between them, and with every feeling of hope and anticipation of success, would close a buoyant paragraph by offering to all those interested in its welfare the compliments of the season. But the stern realities of this eventful period, force us to overlook the amenities of life, and with the earnest wish to do good to our noble profession and still nobler cause, we address ourselves at once to the work before us.

Amid the din of war's wild alarms, when the shock of opposing armies is felt around us, while a new-born nation struggles for its breath, even then the calm, peaceful voice of Science is heard. Let all who love her heed the call. The medical men of the South have had their part to play in the terrible drama. To them have been intrusted the health and well-being of our brave soldiers. Fierce epidemics, spreading fast through crowds of raw recruits, hastily collected, but half prepared for the exposures of camp, and wholly ignorant of the precautions needed to resist disease, have been combatted; an almost total neglect of the laws of hygiene and oftentimes defective nutrition, resulting from improper and badly cooked food, or even a scarcity of the proper articles of diet, have sapped the constitution of the strongest; while the casualties of battle, in a thousand forms, have demanded the most active and immediate surgical interference.

To do justice to the herculean task, to vindicate themselves and their art, the medical staff of our army must fulfil its duty to Science, and prepare to lay before the world the results of its labors. The Journal desires to be the representative of the profession, the exponent of its views, the record of its experience.

Not only as the organ of the Southern medical profession, but as a means of imparting information to those who have, for three years, been debarred from any intercourse with the scientific world, will the publication of a medical periodical be found useful—indeed, an absolute necessity. By taking advantage of the many opportunities to collect the recent literature of medicine, whether as the elaborate treatise or the floating but more practical periodical literature of England and the Continent, the editor hopes to render essential service to his readers, whilst with free access to the archives and reports of the Medical Department, and with the approval and under the supervision of the Surgeon-General, the vast statistics and tabulated records of the war can be carefully collated and made to subserve their legitimate uses.

The task before us is arduous, the difficulties many, but if our brethren will give us an active and steady co-operation, success is not doubtful. We may have the proud satisfaction of believing that something has been done by us to advance and adorn the science to which our lives have been devoted, and with increased opportunities of knowledge we will be better able to do full justice to those whose lives are placed in our charge.

---

*Association of Army and Navy Surgeons.*

At a time when concert of action has been invoked in almost all the departments of the Government for objects of temporary and often doubtful merit, it is gratifying to record, that the head of the Medical Department, equally provident in its executive capacity in establishing order and method in the distribution of official business, and securing such results as grow out of a wisely-conducted and pains-taking administration, has not lost sight of the permanent advantages that might accrue to science out of the progress of this war.

In an earnest and unambiguous zeal for the promotion of our science, and with the judicious foresight which has characterized the vigorous and systematic exertions of the Medical Department, it has enlarged our facilities, and even *created* the means for prosecuting research under most favorable auspices.

Convinced of the great utility of some associated effort among the medical officers of the army, in order to elicit that kind of information which might be said to constitute the individual wealth of the possessor, and therefore not willingly imparted, and certainly not to be voluntarily communicated but through the medium of a mutual interchange of sentiment; reflecting upon the material arrayed in so vast an arena, where observation has long been busied in subjecting speculative inquiry to the rigor of experimental research; and impressed with the belief that by this time we were prepared to speak with authority on most, if not all problematical points, the solution of which would go to form a complete and perfect record of our military surgery, it was thought expedient to convene a meeting of the surgeons of both army and navy, assembled at the capital, who, in debate, and in communication with the field medical officers, might, by the examination of subjects of interest, reach the individual opinions of the medical staff everywhere, and thereby arrive at the expression of a general sentiment as the definite decision upon all such subjects.

Justly appreciating the interest which would be felt in such a project, and the number of contributors who would share in the advantages which its realization would confer, the enterprise was first communicated to those most desirous to promote its successful fulfilment, and then made the subject of a more general deliberation at a meeting sometime since held in this city.

There were many advantages to be obtained from the establishment of a society of this kind, at this particular time, conspicuous among which was co-operation between hospital and field surgeons in the discussion of subjects requiring information from both sources. A provision in the constitution of this association, making corresponding members of those in active service in the field, brings their experience and performances in pertinent relation with that derived from hospital practice, and unfolds results not otherwise reached in all their details, and not exactly to be obtained from the purely statistical records extemporaneously collated amidst moving armies, and for accuracy and completeness, perhaps hopelessly irreconcilable with the accidents of war and confusion of battles.

Private communications addressed to such a body from our field surgeons, while memory is fresh with the most striking events in which they have been participants, are made in all the fullness of easy discussion, untramelled by the provisional limitations within which they would otherwise be restricted in official reports, and are hence rendered far more available for scientific purposes; and it can be little doubted, that invited to this freedom of discussion, much information will be thus secured, which, under the circumstances mentioned, is frequently lost, or the record indefinitely postponed to a more convenient season, when the review of the past is only remembered to lament how much has been forgotten. The latitude given to expression of opinion elicits whatever of novelty may attach itself to the views of particular individuals, and develops the progress reasonably expected, where the ampliest materials are at the command of diligent observers and original thinkers.

The debates which ensue must likewise bring to bear upon the most important subjects under consideration, the enlightenment of minds diversely exercised upon the various phases such topics may present, which would scarcely be examined from as many different points of view by any one individual, when so little time is offered for elaborate investigations or bibliographic review; and this prompt and efficient mode of educing the best and most approved methods of inquiry is not among the least invaluable benefits derived from this public consultation of opinions.

These deliberations also conduce to the promulgation of new discoveries and improvements in our art, not alike accessible to all, since they may be derived from European sources that have reached only some of us, through obstacles and difficulties which a protracted blockade has established.

The repeated meetings of the society serve again to invite attention to a class of facts best illustrated by pathological anatomy, and such specimens as are sometimes exhibited may, probably, lay the the foundation of some subsequent collection as the nucleus of a museum of pathology, in which all the forms of injuries from fire-arms, the peculiar effects of the Minie ball, and interesting processes of repair shall be displayed.

In presence of the diversified labors which such an enterprise suggests, we have reason to congratulate ourselves in announcing the existence, and already active operations, of an organization of the kind.

Indeed, it may be said that the month of August just passed inaugurated a new phase in the aspect of affairs, directly promotive of the great interests of the service. It was during the latter part of August, 1863, under the auspices of the Surgeon-General, that the large number of surgeons of Richmond, through the polite invitation of the Faculty of the Medical College in this city, assembled in one of the halls of that institution to organize an Association of Army and Navy Surgeons of the Confederate States. Upon motion of Professor McCaw, the Surgeon-General was called to the chair, and Surgeon W. A. Davis acted as secretary. At the request of the chairman, Surgeon Middleton Michel stated the objects which convened them on the occasion, and congratulated those present on the large attendance of surgeons, who had so promptly and willingly responded to the call. He observed, that it was the subject of unfeigned gratification, since it gave confidence in the interest likely to be felt in the plan to be proposed, and some degree of assurance as to its ultimate accomplishment. Referring to the experience which the active surgery of more than two years must have developed, he alluded to the skillful interventions of surgical art which had been exhibited, remarking that, perhaps, even such errors as may have been committed, would lead to the establishment of new methods, the instituting of new experiments, which, in their turn, would serve either to communicate information or rectify misconception. Urging the necessity for some consentaneous action, among the surgeons of the Confederate Army, in systematizing their labors and intercommunicating their results, he endeavored to show the expediency of at once establishing an association for the advancement of such an object.

Surgeon J. B. McCaw, in most earnest and fraternal sentiments of regard, seconded these remarks, in welcoming to the hall in which he holds the professorial chair the large delegation of the profession, gathered from distant homes, who have entered upon a pilgrimage in behalf of the interests, the health and comfort of the sick and wounded soldier. He moved that a committee be appointed by the chair to draft a constitution, which being reported upon, was read, discussed and adopted.

Surgeon Garnett moved the appointment of a committee by the chair for the nomination of permanent officers. The same were ballotted for and duly elected. With such changes as have recently occurred through resignations and transfers of position, the present officers of the Association are: Samuel P. Moore, M. D., President; J. B. McCaw, M. D., 1st Vice President; D. Conrad, M. D., C. S. N., 2d Vice President; W. A. Davis, M. D., 1st Recording Secretary; W. A. Thom, M. D., 2d Recording Secretary; M. Michel, M. D., 1st Corresponding Secretary; S. Jenkins, M. D, 2d Corresponding Secretary; and J. S. Wilson, M D., Treasurer.

The debates of this Society have been attended with much interest, and have already eventuated in developing new channels of thought, and, in some respects, disclosed very unexpected and instructive results. Series of questions have for some time been under the consideration of the army surgeons and members of the association, which have been carefully replied to and cautiously considered. Reports of the conclusions arrived at, after weighing the enlightened sentiments of its members and correspondents, may reasonably be supposed to constitute hereafter no inconspicuous portion of the annals of this war. Consonant with this plan, a report of some length was read at the last meeting of the association by Surgeon Michel, embodying a resume of those general considerations on gun-shot wounds contemplated in the first set of questions; in which it was shown that, taking the expression of opinions which have reached us from all portions of the army, both from field and hospital service, it was impossible to determine the orifices of entrance and exit in gun-shot wounds by any *one* feature that may be ascribed to them, though collateral and contingent circumstances would very often adjudge the question. Those characteristics, most frequently referred to as pertainining to these apertures, were shown to be unreliable, for obvious physical reasons which were discursively examined. With regard to the of healing gun-shot wounds by first intention, observations from authentic sources were adduced to justify the conclusion that such an event, even under the destructive influences of an agent like the minie ball, is not impossible. Cases of great interest were mentioned from Surgeons H. F. Campbell, J. B. Read, C. J. Clark, W. S. Mitchell, A. M. Fauntleroy, and others. On the subject of chloroform, a long, instructive and very general discussion arose, in which the invaluable offices of this anæsthetic agent were dwelt upon, especially by Surg. Bolton, while reports read from every battle-field in the Confederacy have served to show the innoxiousness of this potent agent. Where chloroform has been cautiously administered, but one death has been reported which could be justly ascribed to its influences, and in this instance death occurred suddenly. Though various methods of administering anæsthetics, such as have been recommended by Simpson, Snow, and others, and numerous apparatus contrived for the purpose, have been tried, yet no plan has proved safer, better, or more economical than the ordinary mode of presenting it on a small sponge, held at a little distance from the nostrils, and covered by a handkerchief, to prevent too rapid evaporation.

No subject in surgery is more important than that of secondary hæmorrhage, consequent upon gun-shot wounds. This subject, in all its bearings, has claimed much attention, and has especially given rise to views no less novel than strictly scientific, within certain limits. In reference to effects observable in a part after ligation of the main artery for the arrestment of hæmorrhage, Surgeon Campbell furnished additional evidence, on physiological grounds, for considering two very different forms of gangrene, by illustrating the results which followed these operations in limbs in which this morbid condition had commenced and was arrested.

Anticipating communications from our field and hospital surgeons on the subject of primary and secondary hœmorrages, still under consideration, we may infer, from what have already been received, that important reports will, in due time, be specially addressed to the relative value in such instances of local deligation and ligation above the seat of injury.

In accordance with a wish frequently intimated by corresponding members, we here append a copy of the constitution of the association, requesting a continuance of that interest heretofore manifested by those who, even at a distance, have shared in our deliberations, and may be said to have met us in convention through the essays and papers they have so generously communicated:

### CONSTITUTION.

### Article I.

This Association shall be entitled the "Association of Army and Navy Surgeons," and shall have for its object the elucidation of practical and scientific points in the Military Surgery of this war, as illustrated by the individual experience of its members.

Its business shall be transacted at the seat of Government.

ARTICLE II.

Its special design being to increase our information on subjects relating to Medical and Surgical Science by the accumulation of reliable data from the most authentic sources, it is proposed that its members prepare themselves on such points as have particularly engaged their attention, and that they make known their views or opinions to this Association either through the medium of open discussion, or by the reading of papers or essays.

ARTICLE III.

In further fulfilment of this purpose the President will, at one meeting, submit questions on subjects requiring special investigation, the response to which will constitute the principle object of the next meeting, when members will be invited to join in debate, and sustain their views by illustrative cases.

ARTICLE IV.

The papers, essays, or monographs thus furnished, will be handed to the Recording Secretary and will thus constitute the memoirs of the Association.

ARTICLE V.

The aim of this Association being the recognition of the particular opinions of the prominent members of the Medical Staff of the Army and Navy on all subjects contributing to the advancement of Science and alleviation of human suffering, they will be credited with whatever originality is found connected with their researches, by the record of their names in the minutes of the Society, and the careful preservation of their communications.

ARTICLE VI.

Every Surgeon and Assistant-Surgeon of the Medical Staff of the Confederate States Army and Navy may become a member of this Association by signing the Constitution and contributing the sum of ten dollars.

ARTICLE VII.

The Association shall be composed of resident, corresponding, and honorary members.

Correspondents may become members by communicating papers on Medical or Surgical subjects, and by intimating their desire to subscribe to the By-Laws and Constitution.

Honorary members, when duly proposed, shall be duly elected by a majority of the Society present.

ARTICLE VIII.

The officers shall be a President, two Vice-Presidents, two Corresponding Secretaries and two Recording Secretaries.

ARTICLE IX.

The meetings of this body shall be held every fortnight, on Saturday even'ng, at 7 o'clock in winter and at 8 o'clock in summer.

ARTICLE X.

Special meetings may be called by the President on the written application of five members, notice being given a day previous in one or more of the papers of the city, when ten members will constitute a quorum.

ARTICLE XI.

The President shall preside at all meetings, and in his absence one of the Vice-Presidents. He must preserve order, regulate the debates, and previous to adjournment propose questions for discussion at the ensuing meeting.

ARTICLE XII.

The Recording Secretaries shall attend all meetings, and make correct minutes of the proceedings, keeping on file and arranging all papers, essays, &c., read before this Association. They shall, under direction of the President, serve all notices and conduct all other correspondence deemed necessary, and receive all monies paid into the Association. *The special business* of the Corresponding Secretaries will consist in their forwarding, by letter or telegraph, the interrogatories proposed by the Chair to all corresponding members, urging a reply and contributions in the form of papers, essays, etc., etc., which it shall be their duty to read before the Association and hand over to the Recording Secretaries.

ARTICLE XIII.

This Constitution may be altered or amended at any regular meeting by a vote of two-thirds of the members present, provided that written notice of such proposed alteration or amendment shall have been given at three previous meetings.

---

*To Contributors.*

*Original Communications* of merit will always find a welcome place in our columns. Such papers should be brief, pointed and free from any controversial or personal character.

A section of the Journal will always be devoted to *Hospital Reports*. Here, the vast mass of statistics may be slowly and patiently elaborated and placed on record. The interesting subject of compound fracture of femur, and the surgical procedures adopted with results, has been commenced in this number. If hospital surgeons will work actively and *accurately* in this immense field, many interesting deductions will be developed.

A *Chronicle of Medical Science* will be found in each number, collated from recent foreign works and periodicals, which will increase in value and interest as our material enlarges.

Lastly—an *Editorial and Miscellaneous* department will enable the Journal to give much information of general interest in the collateral branches of science.

It will be observed that, instead of a weekly journal in octavo form, a monthly in quarto has been substituted. The uncertainty of the mails and a great economy in paper, with increased amount of reading matter, led to a change of plan in this respect.

All literary matter will be addressed to the Editor; business communications and remittances to the Publishers, Messrs. Ayres & Wade, who will forward the Journal promptly on the receipt of the subscription—*Ten Dollars*—beginning with the first number.

---

*Contents.*

ORIGINAL COMMUNICATIONS........................................ 1

1. Traumatic Tetanus, by Prof. Jos Jones. 2. Resections of the Hip-Joint, by Surg James B. Read. 3. On the External Application of Oil of Turpentine as a Substitute for Quinine in Intermittent Fever, with Reports of Cases. 4 Case of Tetanus—Recovery—reported by Surgeon W. A. Davis.

CONFEDERATE STATES HOSPITAL REPORTS............... 8

EDITORIAL AND MISCELLANEOUS................................ 13

